# PEI-Functionalized Carbon Nanotube Thin Film Sensor for CO_2_ Gas Detection at Room Temperature

**DOI:** 10.3390/mi12091053

**Published:** 2021-08-30

**Authors:** Maeum Han, Soonyoung Jung, Yeonsu Lee, Daewoong Jung, Seong Ho Kong

**Affiliations:** 1 School of Electronic and Electrical Engineering, Kyungpook National University, Daegu 41566, Korea; mehan@knu.ac.kr (M.H.); sallmen@kitech.re.kr (S.J.); yeonsu2629@kitech.re.kr (Y.L.); 2 Advanced Mechatronics R&D Group, Korea Institute of Industrial Technology (KITECH), Yeongcheon 38822, Korea

**Keywords:** carbon dioxide, carbon nanotube, gas sensor

## Abstract

In this study, a polyethyleneimine (PEI)-functionalized carbon nanotube (CNT) sensor was fabricated for carbon dioxide detection at room temperature. Uniform CNT thin films prepared using a filtration method were used as resistive networks. PEI, which contains amino groups, can effectively react with CO_2_ gas by forming carbamates at room temperatures. The morphology of the sensor was observed, and the properties were analyzed by scanning electron microscope (SEM), Raman spectroscopy, and fourier transform infrared (FT-IR) spectroscopy. When exposed to CO_2_ gas, the fabricated sensor exhibited better sensitivity than the pristine CNT sensor at room temperature. Both the repeatability and selectivity of the sensor were studied.

## 1. Introduction

Carbon dioxide is a colorless and odorless gas, contributing to the greenhouse effect; it is a major cause of global warming [1,2,3,4]. Hence, reliable and low-cost CO_2_ gas sensing is of great significance. Since CO_2_ is an unreactive gas, operating gas sensors at room temperature is challenging [5,6]. Diverse sensing principles and/or materials for detecting CO_2_ gas have been reported, including nondispersive infrared (NDIR), metal oxides, polymers, and nanomaterials. Each has their own strengths and weaknesses. Most common sensors are NDIR sensors and metal oxide sensors; however, IR sensors have disadvantages such as bulkiness of transducers and high power consumption during operation [7,8,9,10]. Metal oxide gas sensors typically work at temperatures higher than 200 °C, which increases the difficulty of manufacturing and requires high power consumption [11,12,13]. In contrast, polymer-based sensors do not share these disadvantages. Polymer-based sensors are polymer films that can broadly detect and identify various components in the air to target analyte [14]. A coating of organic detecting layers is easy to synthesize and improves gas detection. In addition, a polymer is easily processable and can be coated using various coating methods such as drop casting and spin coating [15,16,17]. However, polymer receptor layers also are burdened by significant drawbacks, some of which are rapid aging and low resistance to sensor poisoning.

Recently, CNTs have proven to be promising candidates for gas sensitive materials owing to their large effective surface area and abundant sites for adsorbing gas molecules, as well as their hollow geometry, chemical properties, and high aspect ratio. Surface area can promote the physical adsorption or chemical reaction with target gas molecules and efficient and rapid signal conversion [18,19]. The sensing mechanism is based on the transfer of electrons to chemical analytes through charge transfer between the CNTs and gas molecules. As evidenced by experiments involving electron-donating (NH_3_) and electron-withdrawing molecules (NO_2_), the sensing mechanism can still operate where electrons are transferred when interacting with different analytes [20,21].

CNT sensors are sensitive to charge donors and acceptors but not to Lewis acids or bases such as CO_2_ gas [22]. Sensitivity can be improved using a recognition layer that triggers a chemical reaction that alters the properties of the CNT sensor. Although the chemical functionalization of CNTs for the sensor has been explored, the disadvantage of covalent modification is that it destroys the physical properties of CNTs, resulting in a loss of conductance [23,24,25,26]. We performed a noncovalent functionalization of CNTs by applying an amine-rich polyethyleneimine (PEI) polymer. PEI contains primary, secondary, and tertiary amine groups; hence, it can easily adsorb CO_2_ at room temperature [27]. The fundamental mechanism is the interaction between the amino groups with CO_2_ at room temperature to form carbamates by physisorption and reversible reaction between CNTs and CO_2_ gas [28].

CNT sensors can be fabricated using various methods, including direct growth, screen-printing, electrophoresis, and spraying [29,30,31,32]. However, these manufacturing methods are difficult to reproduce, and the sensitivity of the sensors produced will be low. In comparison, CNT thin films prepared using the filtration method can make a dense CNT network. The procedures are simple, including dispersion and filtration of the CNT solution, making them suitable for practical applications. A dense arrangement of CNTs is expected to exhibit higher sensitivity and repeatability. In addition, a CNT thin film with a paper-like appearance is suitable for flexible and conductive applications such as flexible devices [33].

In this study, PEI-functionalized CNT thin film sensors were fabricated for CO_2_ gas detection at room temperature. A highly uniform CNT thin film was used as a resistive network for gas sensing. PEI functionalization was implemented to enhance the CO_2_ capture capability of amino groups. The sensor was characterized through SEM, Raman spectroscopy, and FT-IR, and its responses to different concentrations of CO_2_ gas were studied. In addition, the fabricated sensor was evaluated in terms of its repeatability, selectivity, and flexibility. The results can stimulate research on flexible and wearable gas-sensing systems.

## 2. Materials and Methods

### 2.1. Polyethyleneimine (PEI)-Functionalized Carbon Nanotube (CNT) Thin Film Sensor

**Figure 1:**
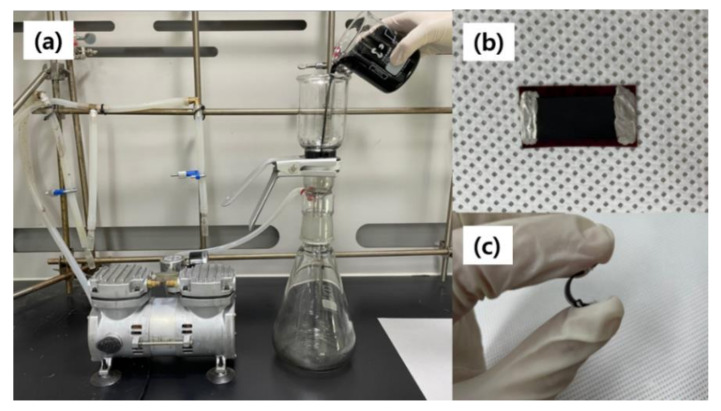
(**a**) Filtration system; (**b**) the image of the fabricated sensor; (**c**) the flexibility of the sensor.

We fabricated a CNT thin film using MWCNTs (Sigma-Aldrich, St. Louis, MO, USA), the average length and diameter of which were in the ranges of 6–13 nm and 2.5–20 μm, respectively. MWCNTs and methanol were mixed in the ratio of 0.2 g per 100 mL, added with 0.5 wt % PEI (Sigma-Aldrich), and the mixture was stirred for 12 h. Thereafter, the mixture was homogenized for 1h using ultrasonic equipment. After fully dispersed, a vacuum filtration process was conducted with 8 μm filter paper (Whatman, Marlborough, MA, USA). After drying the CNT thin film by heating at 70 °C for 1 h, it was placed on a polyimide substrate to make a silver electrode with a gap of approximately 2 cm at both ends of the sample. Subsequently, the silver electrode was heated at 90 °C for 15 min and dried to fabricate a gas sensor. Figure 1 shows the image of the filtration production system, the fabricated sensor, and the flexibility of the sensor.

### 2.2. Gas Sensor Measurement

The gas response of the sensor was measured in a gas chamber at room temperature. The CO_2_ and nitrogen gases (Korea Nano Gas Inc., Hae Song Industry Co., Dongan-gu, Anyang-si, Gyeonggi-do, Korea) were used as target and carrier gases, respectively. During the experiments, the gas concentration was controlled using a mass flow controller (MFC). The total gas flow rate was set to 500 sccm. We used nitrogen gas instead of air as the carrier gas to eliminate the influence of moisture and oxygen in the air. The resistance of the fabricated sensors was sampled while a constant 5 V DC voltage was applied.

## 3. Results and Discussion

### 3.1. Morphology of PEI-Functionalized CNT Thin Film

The morphology of the PEI-functionalized CNT film was studied using SEM. A CNT film is essentially a porous structure composed of randomly entangled CNTs. The filtration method can ensure the uniformity of CNT deposition. The CNT film has a relatively smooth surface, and the roughness ranges from a few hundred nanometers to microns [34]. 

**Figure 2:**
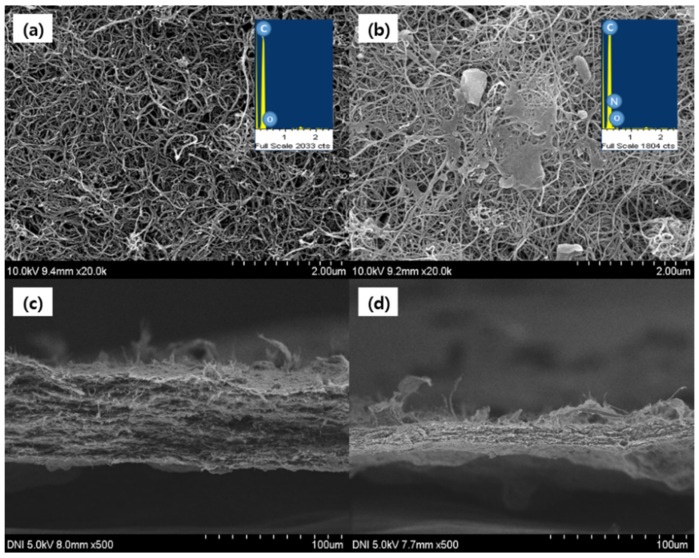
Scanning electron microscope (SEM) images of the top view of (**a**) pristine carbon nanotube (CNT); (**b**) polyethyleneimine (PEI)-functionalized CNT; the side view of (**c**) pristine CNT; (**d**) PEI-functionalized CNT.

Figure 2 shows the morphologies of the pristine and PEI-functionalized CNTs. The diameters of the pristine and PEI-functionalized CNT films are approximately 10 and 16 nm, respectively. As shown in Figure 2b, individual CNTs were wrapped by penetrated PEI into the CNT film, observing the thicker diameter. In addition, the EDS spectrum of the PEI-functionalized CNT emitters exhibited signatures of nitrogen. Because PEI has one nitrogen in every three atoms in the backbone, nitrogen signatures in the PEI-functionalized CNT emitter signifies the presence of PEI at the surface of the CNT [27]. The SEM image shows that PEI was successfully introduced onto the CNTs. 

On the other hand, the thickness of PEI-functionalized CNT film is rather decreased from 60 to 13.5 μm after functionalization of PEI. The outer surface of the CNTs is covered and penetrated by PEI agglomerates because of the viscosity characteristic of the PEI. The PEI and CNT are expected to aggregate and cause a thin thickness of the CNT films. 

### 3.2. Raman Analysis of PEI-Functionalized CNT Thin Film

**Figure 3:**
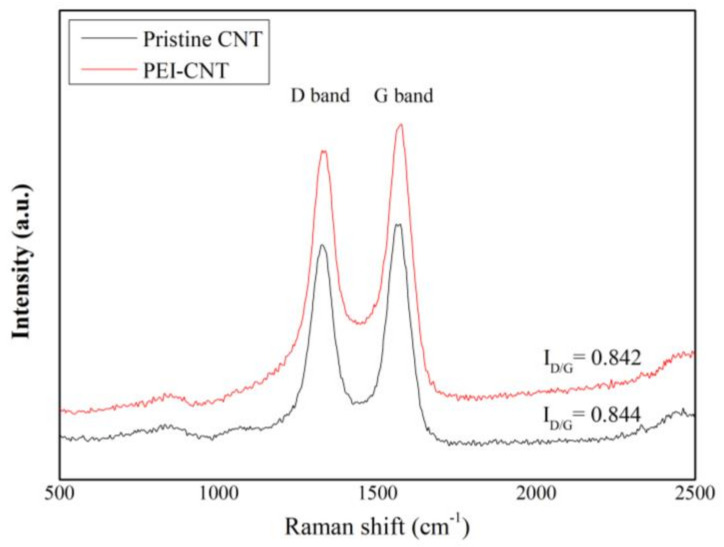
Raman spectroscopy comparison of pristine CNT and PEI-functionalized CNT.

Raman spectroscopy is an effective method for characterizing carbon-based nanomaterials. The fabricated sensor was characterized under a laser power of 10 mW and laser excitation of 532 nm. The effect before and after PEI functionalization of the CNTs was confirmed through Raman spectral observation. Figure 3 shows the Raman spectra measured on raw and PEI-functionalized CNT films. The peaks centered at 1326 and 1571 cm^−1^ represent the D and G peaks of the CNTs, respectively. The shape of the D peak is related to the defect structure of the CNT, and the G band corresponds to the graphite in the lattice. The ratio between the intensities of the D and G bands is denoted by *I_D_/I_G_* value and indicates the degree of CNT defect. 

As shown in Figure 3, the *I*_D_/*I*_G_ values of pristine CNT and PEI-functionalized CNT are similar, which are about 0.842 and 0.844, respectively. These results indicate that PEI functionalization does not significantly damage the CNT structure. For the pristine CNT, the G band was observed at 1571 cm^−1^, but for PEI-functionalized CNT, the G-band showed blue shifted to 1574 cm^−1^. Lu et al. explained that this phenomenon was caused by that the lone pair electrons of N atoms in chains interact π-electrons of CNT. Therefore, the electron density of CNT was strengthened, and polarization of CNT was increased due to the p–π conjugation of the CNT [35,36].

### 3.3. Fourier Transform Infrared (FT-IR) Analysis of PEI-Functionalized CNT Thin Film

**Figure 4:**
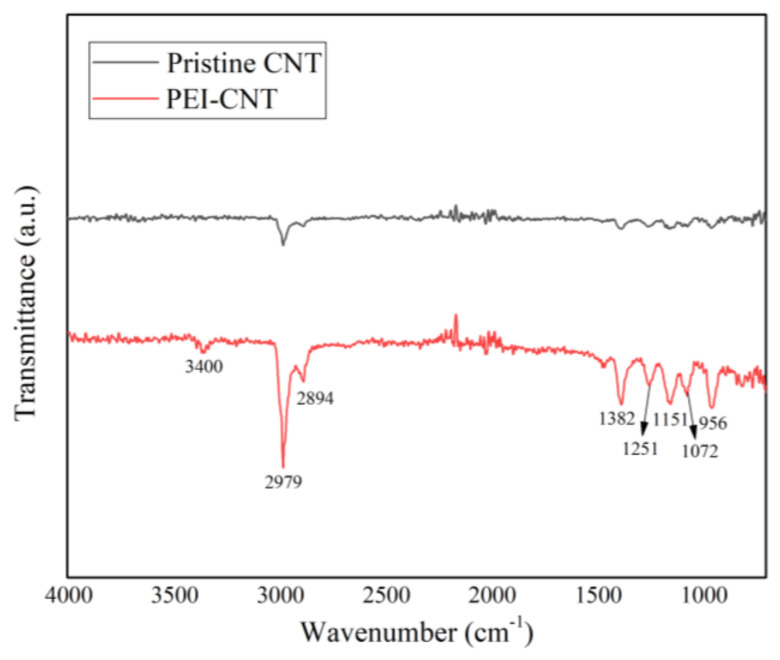
Fourier transform infrared (FT-IR) spectroscopy comparison of pristine CNT and PEI-functionalized CNT.

FT-IR analysis was carried out the analysis of PEI-functionalized CNTs in comparison with the pristine CNTs. Figure 4 shows the FT-IR spectra of the pristine and PEI-functionalized CNTs. The organic moieties of PEI clearly appear themselves in the region at 2800–3000 cm^−1^ (C-H stretching) in contrast with pristine CNTs. In addition, a new band at 3401 cm^−1^ (N-H stretching) appears, and the band at 1382 cm^−1^ and 1072 cm^−1^ also correspond to the C-N stretching vibration due to the PEI [37,38]. From the results, it can be confirmed that the amine group was successfully introduced on the surface of the CNT thin film.

### 3.4. Sensor Responses

The adsorption/desorption of gas molecules has a significant effect on the electrical properties of CNTs. It has been reported that the CO_2_ gas sensing of pristine CNTs is low because of the weak interaction between CO_2_ molecules and the CNT surface [22]. However, it has been found that the sensitivity of CNTs to CO_2_ gas can be significantly increased through surface functionalization [39,40]. To confirm the CO_2_ gas-sensing properties of CNTs, both pristine and functionalized CNT sensors were fabricated, and gas-sensing experiments were conducted.

**Figure 5:**
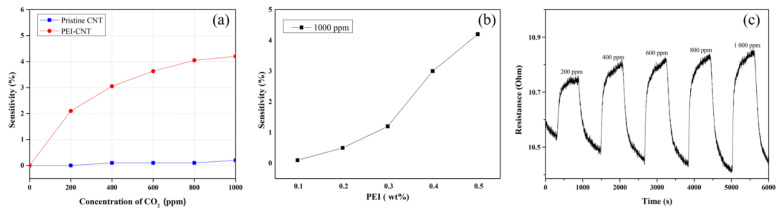
The sensitivities of (**a**) pristine- and PEI-CNT sensors; (**b**) different ratio of PEI; (**c**) the CNT sensor to the concentration of CO_2_ gas from 200 to 1000 ppm.

To evaluate its response to CO_2_ gas, the sensor was placed in a chamber, and the flow rate of the mixed gas was set to 500 sccm. The sensitivity to the CO_2_ concentration was measured and determined using the following equation:

(1) $$\mathrm{Response}\;\left(\%\right)=\frac{\left(R-R_{0}\right)}{R_{0}}\times 100,$$

where *R* is the change in the resistance of the sensor after gas injection, and *R*_0_ is the resistance of the gas sensor before the reaction. Figure 5 shows the gas-sensing characteristics of the pristine and PEI-functionalized CNT sensors. When the sensor was exposed to CO_2_ gas, the electrical resistance of the PEI-functionalized CNT sensor increased with the increase in the CO_2_ gas concentration.

Typically, CO_2_ is a nonreactive molecule, but it combines with an amine group to form a carbamate. This combination can be explained based on the hard soft acids bases (HSAB) theory. The HSAB theory can explain the direct interaction between the CO_2_ molecules and the amino group-based polymer. Hard Lewis acids tend to bind hard Lewis bases, and soft Lewis acids tend to bind soft Lewis bases. Since CO_2_ is a hard acid, it can interact with the primary amines in PEI, which are a hard base. PEI is a highly branched polymer with primary, secondary, and tertiary amino groups. At room temperature, the reaction of an amino group with CO_2_ is reversible, forming carbonate [41].

According to the HSAB theory, the test results are appropriate. In addition, PEI exhibits n-type properties because of the electron-donating properties of the NH_2_ group in PEI [5,22]. The reaction between the CO_2_ molecule and PEI forms carbamates, which reduce the overall electron-donating effect of PEI. Thus, the PEI exhibits properties consistent with electron removal from the sensor. The high density of the amine functional groups in PEI is associated with significant n-doping to the point where there is an adverse effect of p-doping [42]. Therefore, the electrical resistance of the fabricated sensor showed a characteristic that increased with the introduction of CO_2_ gas. Figure 5 shows the CO_2_ gas sensitivity, different ratio of PEI, and response time of pure and PEI-functionalized CNTs. The CO_2_ gas concentration was measured while varying it from 200 to 1000 ppm. As shown in Figure 5a, the reactivity of pure CNTs to CO_2_ gas at room temperature is low; however, the PEI-functionalized CNT sensor exhibits a greater reactivity to CO_2_ gas. After the CO_2_ gas was injected into the chamber, the resistance of the sensor increased rapidly until it stabilized, and the resistance change increased linearly with increasing CO_2_ gas concentration. When the CO_2_ gas injection was stopped and only nitrogen was injected into the chamber, the resistance of the sensor returned to its initial value. Moreover, higher sensitivity was observed in the CNT sensor with a large amount of PEI, as shown in Figure 5b. A larger PEI:CNT ratio can provide more active sites for adsorbed CO_2_ gas. This result was in line with above sensing mechanism. Subsequent experiments were conducted using a sample with 0.5 wt % of PEI.

The response time *T_a_* was defined as the time required for the change in the resistance to reach 90% of the equilibrium value after injecting the target gas, and the recovery time *T_b_* was defined as the time required for the sensor to return to the initial 10%. The sensor was exposed to CO_2_ gas until near saturation was reached, the CO_2_ injection was stopped, and the sensor was desorbed by injecting only nitrogen gas until the resistance returned to its initial value. Figure 5c shows that the response and recovery times of the sensor are the same, i.e., approximately 10 min. After adsorbing the CO_2_ molecules, the PEI acted as a mediator between the CNTs and CO_2_ gas, facilitating charge transfer. Because of the interaction between PEI and the adsorbed molecules during desorption, electrons can be effectively removed from the CNTs without the use of an external source, such as external heating. Moreover, because of the dense CNT thin film structure, it enables stable electron transport to the electrode when measuring the resistance.

**Figure 6:**
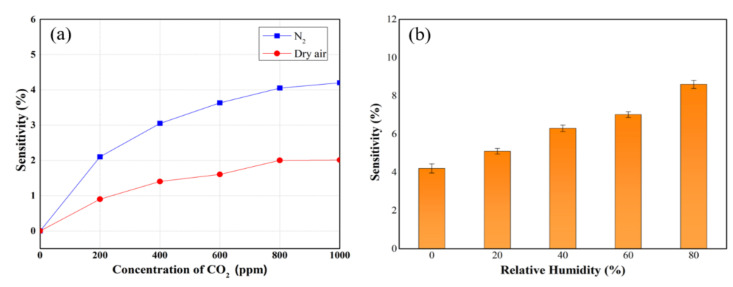
The sensitivities of PEI-functionalized CNT sensors under (**a**) nitrogen and dry air; (**b**) different humidity level.

To evaluate the actual ability of the sensor to operate, the comparison experiments were conducted in dry air as carrier gas instead of nitrogen gas and different relative humidity (RH) values. As shown in Figure 6a, the sensitivity of the PEI-functionalized CNT sensor is reduced under dry air environmental conditions. The degraded performance in dry air is due to the fact that a compensation of n-doping effect by reacting oxygen (O_2_) gas in dry air with PEI on CNT films [42]. On the other hand, upon exposure of the PEI-functionalized CNT sensor in humid nitrogen gas, the sensitivity remarkably increased, reaching an increase of 2 times at 1000 ppm of CO_2_ in 80% RH, as shown Figure 6b. These results are same as those of previous studies [43,44]. Son et al. reported that the charge transfer between PEI and CNTs plays an important role in the improvement of sensitivity under wet conditions. They proposed that the functionalization of PEI on CNT generated the n-doping effect, which encourages the donation of electrons from the protonated polymer, leaving the water molecules in the humidity to act as a source of protons. Once CO_2_ gas was introduced to the sensor, the charge movements occurred between the amino group in the PEI and the CO_2_ gas to result in an acid–base equilibrium state, supporting the formation of carbamates and bicarbonates [43]. Yoon et al. also explained that the exposure to CO_2_ gas under a humidified atmosphere generates amidinium bicarbonates, which produce an increase in the density of mobile hole carriers in the polymer chain, which in turn increases the overall conductance of the sensor and leads to a response [44].

**Figure 7:**
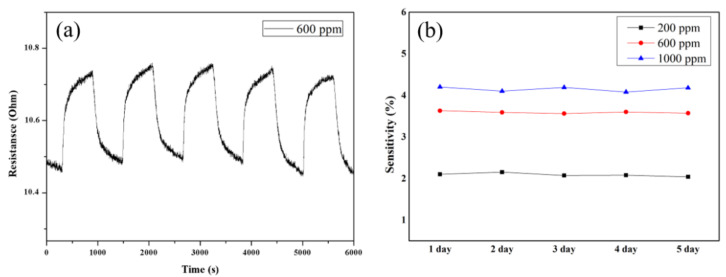
The (**a**) repeatability and (**b**) stability of PEI-functionalized CNT sensor.

To confirm the repeatability and stability of the sensor, five CO_2_ gas response characteristics and long-term experiments were performed. As shown in Figure 7a, a stable and reversible reaction characteristic can be observed in several repeated experiments, with negligible hysteresis. As described above, the CNT thin film prepared using the filtration method is constituted by stacking a large number of dense individual CNTs. Because of the strong van der Waals force and large contact area between the stacked CNTs, the stability was high even after repeated experiments, which prevented damage. In comparison, in the case of printed or spray-based CNT sensors, the low density of individual CNTs and weak interactions result in structural damage after repeated experiments, thereby deteriorating the sensor properties [45]. In terms of repeatability, the experiment was performed five times with a CO_2_ gas concentration of 600 ppm, a gas adsorption time of 10 min, and a desorption time of 10 min, with all the cycles lasting 20 min. The results showed that the CO_2_ gas detection characteristics have good reproducibility and stability as shown Figure 7b.

**Figure 8:**
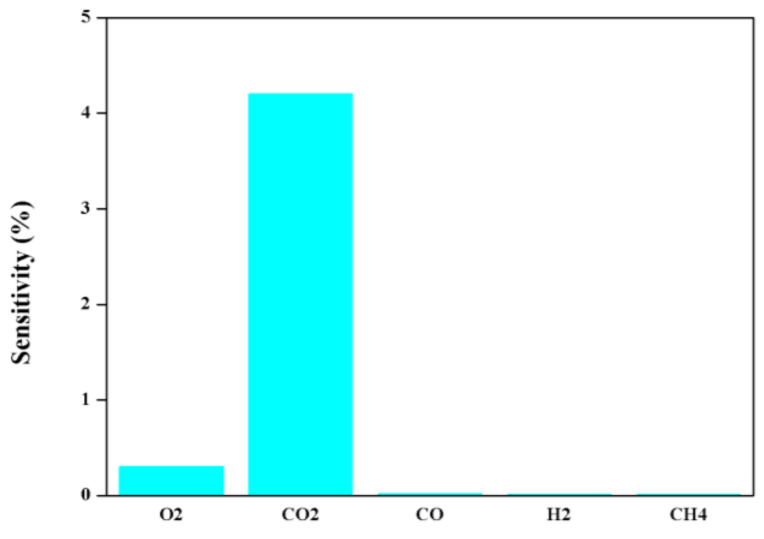
The selectivity of PEI-functionalized CNT toward various gases.

The selectivity of the sensor for a specific gas is an important characteristics of the sensor. Selectivity is the capability of a sensor to measure only one gas species relative to other gases. Typically, CNTs react with other gases such as NH_3_ and NO_2_ at room temperature [46,47]. Therefore, it is particularly important to improve the CNT-based CO_2_ gas selectivity of the sensor for commercialization. Figure 8 shows the sensitivity of the PEI-functionalized CNT gas sensor to different gases. The sensitivity to CO_2_ gas is highest (4.2%) at 1000 ppm. O_2_ gas reacted slightly with the sensor, but PEI interacted more strongly with the CO_2_ gas at room temperature; thus, the reaction value was much lower than that of the CO_2_ gas.

With the growing demand for wearable and flexible device products, the demand for flexible sensors is increasing. These flexible sensors require properties that are flexible but not volatile with respect to sensitivity and shape. However, in the case of a metal or metal oxide-based sensor, it is difficult to fabricate a flexible sensor, but in the case of a sensor using a CNT thin film, a high flexibility can be obtained [48].

**Figure 9:**
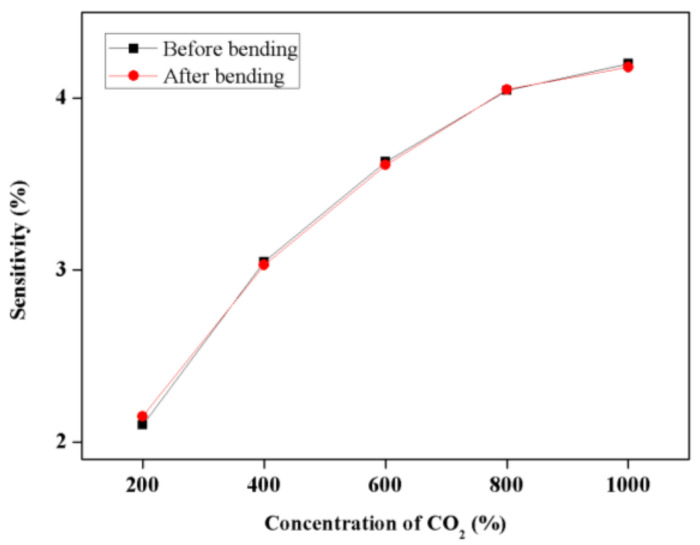
The sensitivity of PEI-functionalized CNT before and after bending tests.

To evaluate the flexibility of the fabricated sensor, the sensor was fabricated on a polyimide film, bent and unfolded several times in a cylinder with a radius of 8 mm, and then the sensitivity characteristics based on the CO_2_ gas concentration were evaluated. After the bending test, no change in the shape of the sensor was observed, and it was confirmed that the resistance was largely the same. Figure 9 shows the sensitivity results with respect to the CO_2_ gas concentration before and after bending. The fabricated sensor has structural stability and flexibility owing to the very high density of the CNT film, and its applicability to wearables and portable devices was confirmed. 

The Table 1 shows a comparison of the sensing properties of reported CO_2_ sensors. The PEI-functionalized CNT sensor was successfully created for sensitive, responsive, and flexible measurement of CO_2_ over a wide concentration range at room temperature.

## 4. Conclusions

In summary, a PEI-functionalized CNT sensor was fabricated for CO_2_ gas detection at room temperature. A PEI-functionalized CNT thin film was fabricated using the filtration method. The response performance of the sensor to CO_2_ gas at room temperature was investigated. The sensor was found to exhibit better sensing characteristics than the pristine CNT sensor because of the acid–base interaction between the amine groups in PEI and CO_2_ gas molecules. The proposed sensor shows characteristics such as good sensitivity, repeatability, and selectivity. In addition, the sensor exhibits good flexibility owing to the high density of the CNT film. These results confirm its potential for wearable and portable CO_2_ devices.

## Figures and Tables

**Table 1:** Comparison of the sensing properties of CO_2_ sensors.

Materials	Type of Sensor	Gas Concentration (ppm)	Sensitivity (ΔR/R)	Operating Temperature (°C)	Flexibility	Reference
ZnO	Chemiresistor	200–1025	2.3	250	-	[49]
Porous silicon/α-MoO_3_	Chemiresistor	50–150	15	250	-	[50]
PEDOT-BPEI	Chemiresistor	1000	2.7	Room tem.	-	[51]
PIL-Al_2_O_3_	Chemiresistor	150–2400	1.7	Room tem.	-	[52]
CNT	Chemiresistor	50–800	2.25	Room tem.	Flexible	[53]
PEI-CNT	Chemiresistor	200–1000	4.2	Room tem.	Flexible	This work

## Data Availability

The data presented in this study are available on request from the corresponding author.
